# Prevalence of nontuberculous mycobacteria in bronchiectasis: A systematic review and meta-analysis

**DOI:** 10.1016/j.nmni.2026.101781

**Published:** 2026-06-06

**Authors:** Mosayeb Rostamian, Shirin Bavari, Nabi Jomehzadeh, Maryam Haddadzadeh Shoushtari, Ebrahim Kharazinejad, Amir Zahedi, Arezo Barani, Milad Abdi, Sara Kooti

**Affiliations:** a Infectious Diseases Research Center, Health Policy and Promotion Institute, Kermanshah University of Medical Sciences, Kermanshah, Iran; b Department of Microbiology, School of Medicine, Iran University of Medical Sciences, Tehran, Iran; c Department of Microbiology, Faculty of Medicine, Ahvaz Jundishapur University of Medical Sciences, Ahvaz, Iran; d Air Pollution and Respiratory Diseases Research Center, Ahvaz Jundishapur University of Medical Sciences, Ahvaz, Iran; e Department of Anatomical Sciences, Faculty of Medicine, Abadan University of Medical Sciences, Abadan, Iran; f Student Research Committee, School of Medicine, Kermanshah University of Medical, Sciences, Kermanshah, Iran; g Department of Medical Basic Sciences, Shoushtar Faculty of Medical Sciences, Shoshtar, Iran; h Infectious Ophthalmologic Research Center, Ahvaz Jundishapur University of Medical Sciences, Ahvaz, Iran

**Keywords:** Nontuberculous mycobacteria, Bronchiectasis, Lung diseases, Prevalence

## Abstract

**Background:**

Nontuberculous mycobacteria (NTMs) are widely distributed environmental microorganisms. NTMs play a significant role in causing respiratory infections, especially in individuals with underlying lung diseases such as noncystic fibrosis bronchiectasis. NTMs are recognized as significant pathogens in bronchiectasis, among other infectious agents. Therefore, this systematic review aims to address the prevalence of atypical mycobacteria in bronchiectasis.

**Methods:**

A search was conducted through the electronic databases PubMed, Web of Science and Scopus via appropriate keywords. After the studies that did not qualify were excluded, the studies with inclusion criteria according to the PRISMA checklist were used to extract the desired information.

**Results:**

The overall prevalences of NTM and NTM-PD were 0.158 (95% CI: 0.084, 0.276) and 0.158 (95% CI: 0.067, 0.328), respectively, according to the results. A variety of species or complexes were examined in the included research, with the majority of them using MAC. *M. avium complex* (MAC) had the highest frequency (0.094, 95% CI: 0.045, 0.185), whereas *M. kansasii* had the lowest frequency (0.008, 95% CI: 0.005, 0.012).

**Conclusions:**

The findings of the present systematic review demonstrate the increasing importance of NTM, particularly MAC, as a significant pathogen in bronchiectasis.

## Graphical abstract

**Figure:**
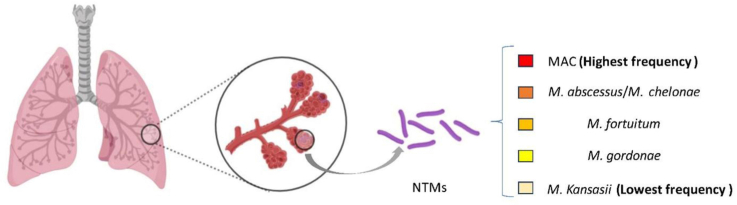

## 1 Introduction

Nontuberculous mycobacteria (NTMs) are widespread environmental microorganisms. They are commonly found in various environmental reservoirs, including soil and water, and serve as potential sources of infection. NTM can occasionally cause respiratory disease, particularly in individuals with compromised immune systems or underlying pulmonary disease [1,2]. The environment is thought to be the primary reservoir for NTM infections, as human-to-human or human-to-animal transmission is rare [3]. NTMs play a significant role in causing respiratory infections, especially in individuals with underlying lung diseases such as noncystic fibrosis bronchiectasis [4].

In patients with bronchiectasis, the bacterial species most frequently isolated from their sputum culture are *H. influenzae*, *P. aeruginosa*, *M. catarrhalis*, and *S. aureus*. With documented prevalence rates ranging from 1% to 18%, NTMs are recognized as important pathogens in bronchiectasis, among other infectious agents [5]. Several species, including *Mycobacterium avium*, *Mycobacterium fortuitum,* and *Mycobacterium chelonei,* have been isolated from environmental samples, including soil and water [6]. The most common bacteria responsible for NTM lung infections are members of the *Mycobacterium abscessus* complex and the *Mycobacterium avium* complex (MAC) [7]. Persistent *Mycobacterium abscessus* infection is bothersome and associated with a significant decline in lung function. In addition, the infection's high level of innate antibiotic resistance makes it very difficult to eradicate [8]. Owing to advances in diagnostic methods, the number of NTM isolates identified has increased in recent years [9]. Individuals with chronic lung disease are particularly susceptible to NTM disease [10]. The clinical significance of NTM in non-CF bronchiectasis (NCFB) has been increasingly recognized in recent decades, as evidenced by the increase in reported NTM lung infections [11].

Bronchiectasis, a chronic lung disease, is defined by irreversible dilatation of the bronchi. Serious complications of this condition include chronic productive cough, airflow obstruction, shortness of breath, and recurrent infections [12]. An initial event often disrupts mucociliary clearance of the bronchial tree. Bacterial colonization impairs ciliary function and stimulates an immune response that exacerbates lung damage [12,13]. Although the association between bronchiectasis and NTM lung disease is well established, the mechanism underlying this association remains unclear. Before an NTM infection, bronchial anatomical alterations are likely to occur [14]. Destruction of cartilage and smooth muscle by MAC is proposed to lead to bronchiectasis, with granulomas indicating established disease after chronic infection [10].

The number of individuals who are susceptible to mycobacterial disease, e.g., due to immunosuppression, is increasing [15]. Research has shown that the prevalence of NTM in bronchiectasis is increasing, and investigating the type of NTM and the geographical area concerning the frequency of NTM in bronchiectasis is crucial [[16], [17], [18]]. Therefore, this systematic review aims to address the prevalence of atypical mycobacteria in bronchiectasis.

## 2 Methods

### 2.1 Search strategy and study selection

A comprehensive search was performed across three major databases—Web of Science, Scopus, and PubMed—using the following keywords: “atypical Mycobacteria,” “nontuberculous Mycobacteria,” “NTM,” and “bronchiectasis,” either individually or in combination with the ‘‘AND’’ and ‘‘OR’’ operators. The PRISMA checklist for diagnostic test accuracy was employed to assess the retrieved articles [19], and their quality was evaluated via the Joanna Briggs Institute scoring system. All studies were screened stepwise, beginning with the title and abstract, followed by a review of the full-text articles.

The detailed search strategy applied to each database, including the specific Boolean logic and field identifiers, is provided in Table S1. Sources such as grey literature, preprint servers, clinical trial registries, and manual reference list screening were not included in the search process.

### 2.2 Inclusion/exclusion criteria

The inclusion criteria consisted of cross-sectional and case‒control studies published as original articles on "nontuberculous mycobacteria" in combination with "bronchiectasis." Studies that did not meet the inclusion criteria, such as cohort studies; letters to the editor; narrative or systematic reviews; studies lacking appropriate data; and non-English articles, conference abstracts, and dissertations, were excluded.

### 2.3 Data extraction

Data from eligible articles were extracted, including the following variables: first author, year of publication, country, study duration, percentage of females, percentage of males, smoking status, mean age of patients, number of patients, number of controls, number of NTM cases, number of NTM-PD cases, isolated species, NTM definition guidelines, detection methods, further identification methods, and gene detection methods.

### 2.4 Data analysis

The meta-analyses were conducted via Comprehensive Meta-Analysis V2.2.064 to evaluate the frequency of NTM and NTM-PD cases, as well as the prevalence of various bacterial species. The frequencies are expressed with 95% confidence intervals (CIs) to provide an estimate of the precision of the results. The choice between fixed effects or random effects models for each parameter was made on the basis of the level of heterogeneity observed across the studies. To investigate potential sources of heterogeneity related to geographical differences, a subgroup analysis by country was conducted. Heterogeneity among studies was quantified via I-squared (I^2^) statistics and assessed for statistical significance via the Cochrane Q test. Owing to the absence of a suitable method for evaluating potential publication bias in frequency studies [20], no assessment of publication bias was conducted in this study. For all analyses, a p value of less than 0.05 was considered the threshold for statistical significance.

## 3 Results

### 3.1 Literature search results

**Fig. 1:**
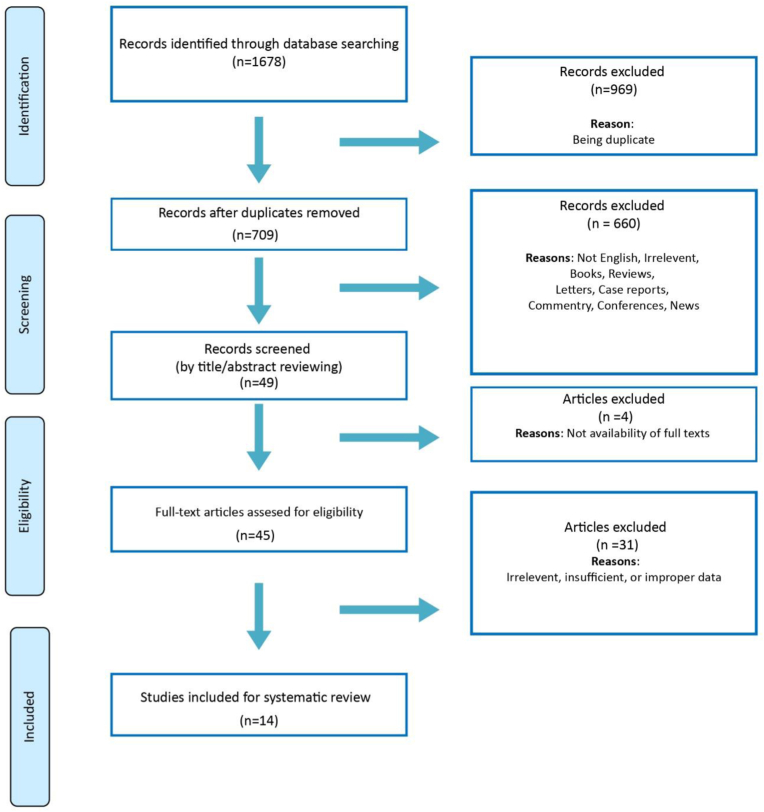
PRISMA flow diagram of study selection.

The database searches revealed 1678 research studies, of which 969 were discarded as duplicates. For the grounds specified in our inclusion/exclusion criteria, 660 of the 709 remaining articles were deemed ineligible. Forty-nine and forty-five studies remained after accurate title/abstract and full-text reading, respectively. After 31 studies were removed because of inadequate data reporting, 14 papers remained for the meta-analysis (Fig. 1).

### 3.2 General data

The first published study included in this review was in 2008, and since then, the number of studies has increased, particularly in recent years. The majority of studies were conducted in China (3 studies), Israel (3 studies), Egypt (2 studies), and Taiwan (2 studies), with one each from Germany, Italy, South Korea, and the USA (Table 1).

**Table 1:** Characteristics of studies.

Authors	Year	Country	Duration of study	Female (%)	Male (%)	Smoking No. (Yes/No)	Mean age patients	Bronchiectasis patients No.	NTM No.	NTM - PDNo.	Isolated species (No.)	NTM definition guidline	Detection method	Further identification methods	Gene detection	Reference
Brown-Elliott et al.	2019	USA	2017-2018	N/A	N/A	N/A	N/A	116 patients (297 samples)	181	N/A	*M. abscessus* (129), MAC (28), *M. chelonae* (1), *M. mucogenicum* (1), *M. gordonae* (3), *M. paraffinicum* (1)	N/A	Culture	VersaTrek	*rpo*B/16S rRNA	[21]
Darwish et al.	2020	Egypt	2017-2018	52.5	47.5	N/A	55.2	40	3	N/A	*M. kansasii* (1), MAC (2)	N/A	PCR	PCR	N/A	[22]
Dettmer et al.	2021	Germany	2011-2020	60	40	N/A	N/A	128	N/A	36	MAC (32), *M. abscessus* (2), *M. kansasii* (2)	ATS/IDSA	Culture	Radiology	N/A	[23]
Eisenberg et al.	2020	Israel	2008-2018	59.9	40.2	N/A	66.5	242	N/A	32	*M. simiae* (11), *M.intracellulare* (8), *M. fortuitum* (7), *M. avium* (2), *M. chelonae* (2), *M. Kansasii* (2), *M. abscessus* (1), *M. xenopii* (1) and *M. gordonae* (1)	N/A	Clinical, laboratory and radiological data and available CT scans	N/A	N/A	[24]
Elmongi et al.	2024	Egypt	2021-2022	46	54	66 (No)/22 active smokers/12 ex-smokers	52.11	100	7	N/A	*M. kanssasii* (2), Other NTM (5)	ATS	Culture	Biochemical tests/PCR	16S rRNA/*ITS*	[25]
Faverio et al.	2016	Italy	2012 - 2015	58	42	N/A	N/A	141	32	23	MAC (24), *M. gordonae* (4), *M.chelonae* (2), *M.kansassii* (1), *M. shimoidei* (1), *M.abscessus* spp. (1)	ATS/IDSA	Culture	Radiology	N/A	[26]
Feng et al.	2023	China	2021-2023	47	53	64 (No)/22 (Yes)	N/A	86	N/A	34	N/A	ERS/BTS	Culture	Sequencing	16S rRNA/*hsp*65	[27]
Frajman et al.	2024	Israel	2007-2020	61.4	38.6	232 (Prior or current somking)	61.11	771	N/A	94	N/A	N/A	Culture	GenoType Mycobacterium DNA strip assay	N/A	[28]
Guang-suo et al.	2008	China	2001-2006	N/A	N/A	N/A	37	105	7	N/A	N/A	N/A	Culture	N/A	N/A	[29]
Hsieh et al.	2018	Taiwan	2005-2014	53	47	19 (Yes)	65.3	96	35	N/A	MAC (18), *M. fortuitum* (8), *M. chelonae* (8), *M. abscessus* (5), *M. gordonae* (4), *M. kansasii* (1), *M. mageritense* (1), *M. scrofulaceum* (1), *M. peregrinum* (1)	ATS/ERS	Culture	N/A	N/A	[30]
Kuint et al.	2024	Israel	2012-2017	N/A	N/A	N/A	N/A	293	15	N/A	N/A	N/A	Culture	Radiology	N/A	[31]
Kwak et al.	2020	South Korea	2011-2019	38.9	61.1	N/A	N/A	221	35	31	*M. avium* (17), *M. intracellulare* (8), *M. abscessus* (2), *M. avium* and *M. intracellulare* (3), *M. avium* and *M. massiliense* (1)	ATS/IDSA	Culture/AFB/radiography	N/A	N/A	[32]
Wang et al.	2024	Taiwan	2017-2020	58.3	41.7	665 (yes)/1949 (No)	66.4	2614	N/A	79	*M. abscessus* complex (34), MAC (30), *M. kansasii* (10)	ATS/ERS/ESCMID/IDSA	Culture	CT	N/A	[33]
Yin et al.	2021	China	2018-2020	66	34	17 (current)/20 (second-hand smoke)	N/A	202	N/A	47	MAC (31), *M. abscessus*/*M. chelonae* complex (13), *M.kansasii* (3)	ATS	Culture	Radiology	N/A	[34]

**Abbreviations:** AFB: acid fast bacilli, ATS: american thoracic society, BTS: british thoracic society, ERS: european respiratory society, ESCMID: european society of clinical microbiology and infectious diseases, IDSA: infectious diseases society of america, MAC: *Mycobacterium avium* complex including *M.avium*, *M. intracellulare and M. chimaer*, NTM: nontuberculous mycobacteria, NTM-PD: non-tuberculous mycobacterial pulmonary disease, PCR: polymerase chain reaction, *rpo*B gene: codes RNA polymerase β subunit,16s rRNA: 16S ribosomal RNA, N/A: not available.

The included cases included both genders and various age groups, as well as individuals with different smoking statuses. Owing to the variability of these factors, statistical analyses could not be performed to assess their effects (Table 1).

The most commonly used guidelines for defining NTM cases were those of the American Thoracic Society (ATS) (7 records), the Infectious Diseases Society of America (IDSA) (4 records), and the European Respiratory Society (ERS) (3 records). The primary detection methods included bacterial culture (12 studies). Genetic analysis was performed in only three studies, with the detected genes including 16S rRNA, *hsp*65, *rpo*B, and *ITS* (Table 1).

### 3.3 Frequencies of NTM and NTM-PD in bronchiectasis patients

**Fig. 2:**
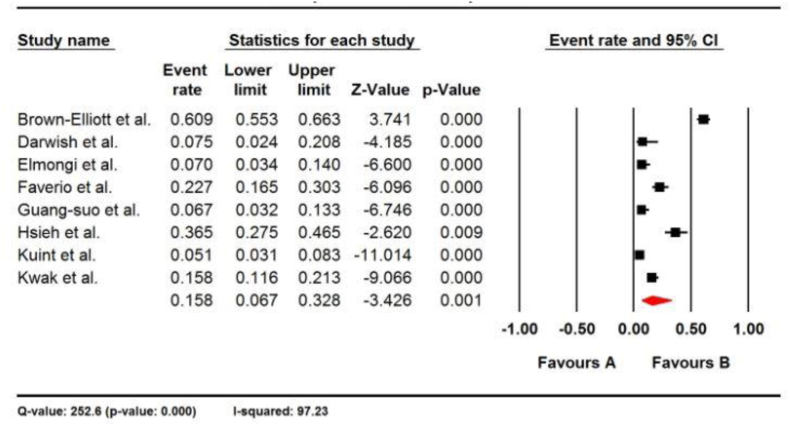
NTM prevalence in bronchiectasis patients.

Eight studies reporting the number of bronchiectasis patients or samples and the presence of NTM isolates were included in the meta-analysis. The findings revealed that the overall prevalence of NTM was 0.158 (95% CI: 0.067, 0.328). Significant heterogeneity between studies was observed, with a Q value of 252.6 (p value: 0.000) and an I^2^ of 97.23% (Fig. 2). Importantly, subgroup analysis by country was not performed because there are few (one or two) studies in each country.

**Fig. 3:**
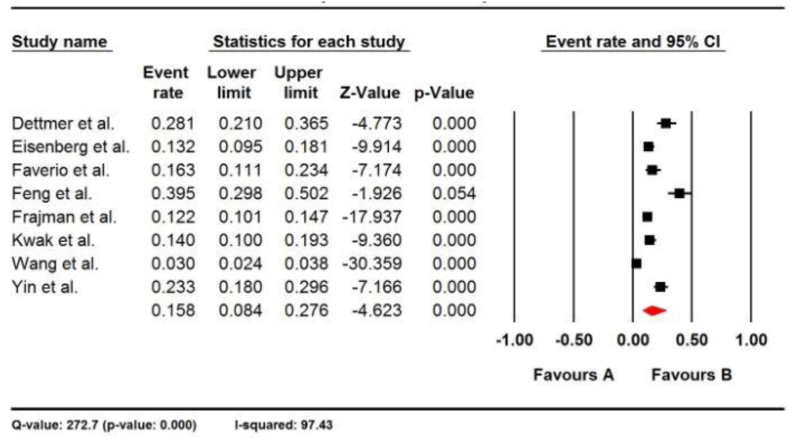
NIM-PD prevalence in bronchiectasis patients.

Eight studies reporting NTM-PD cases were included in a meta-analysis to assess the prevalence of NTM-PD. The analysis revealed an overall prevalence of NTM-PD of 0.158 (95% CI: 0.084, 0.276). Significant heterogeneity was observed across the studies, with a Q value of 272.7 (p value: 0.000) and an I^2^ of 97.43% (Fig. 3).

### 3.4 Frequency of species in bronchiectasis patients

**Fig. 4:**
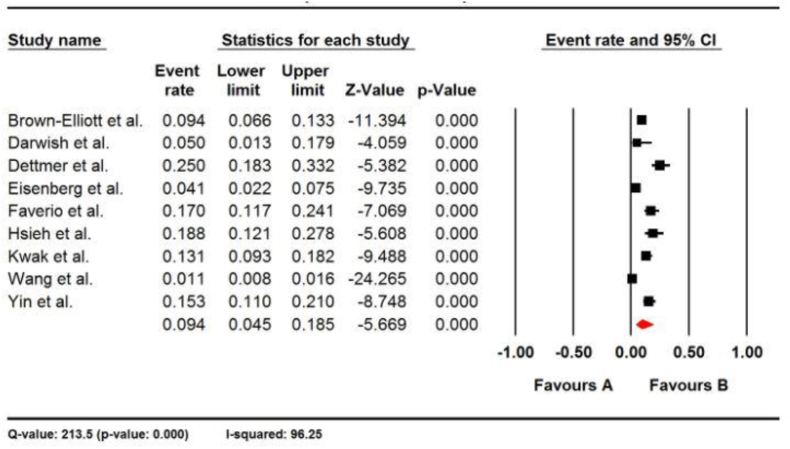
MAC prevalence in bronchiectasis patients.

**Fig. 5:**
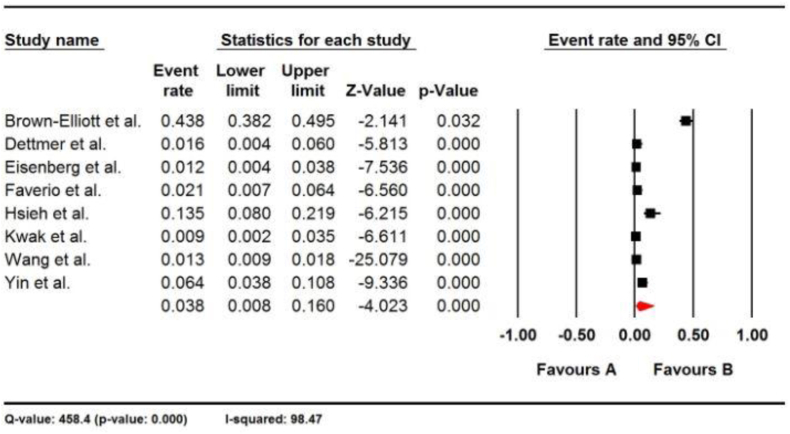
*M. abscessus*/*M. chelonae* prevalence in bronchiectasis patients.

**Fig. 6:**
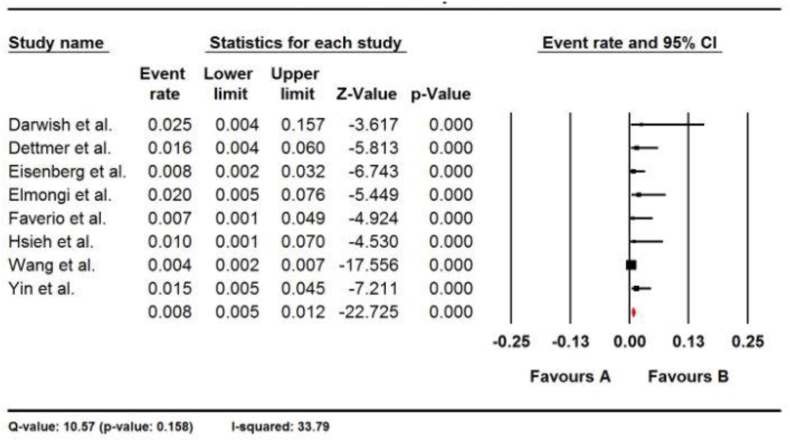
*M. kansasii* prevalence in bronchiectasis patients.

**Fig. 7:**
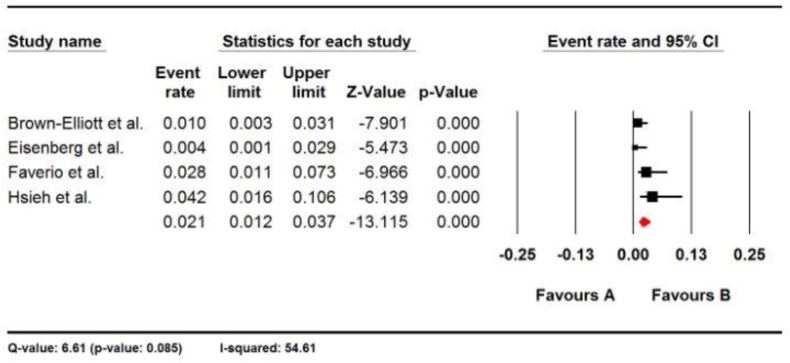
*M. gordone* prevalence in bronchiectasis patients.

**Fig. 8:**
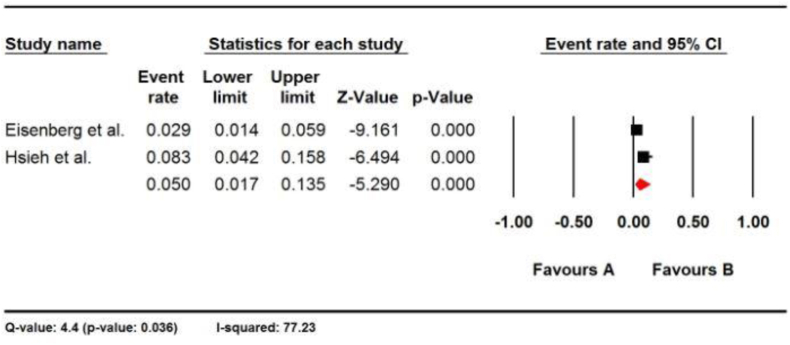
*M. fortuitum* prevalence in bronchiectasis patients.

In the included studies, a total of several species or complexes were investigated, with most cases of MAC (9 records), *M. abscessus*/*M. chelonae* (8 records), *M. kansasii* (8 records), *M. gordonae* (4 records), and *M. fortuitum* (2 records) (Table 1). The frequencies of these species in bronchiectasis patients were analyzed (Fig. 4, Fig. 5, Fig. 6, Fig. 7, Fig. 8). The highest frequency was observed for MAC (0.094, 95% CI: 0.045, 0.185) (Fig. 4), whereas the lowest frequency was recorded for *M. kansasii* (0.008, 95% CI: 0.005, 0.012) (Fig. 6).

## 4 Discussion

Currently, the prevalence of NTM infection is rising globally [35]. People with lung conditions such as bronchiectasis are particularly susceptible to it [36]. According to the guidelines set forth by the European Respiratory Society (ERS) and the British Thoracic Society (BTS), people with bronchiectasis should be screened for NTM [37,38]. This study thoroughly examined the prevalence of NTM infection in patients with bronchiectasis. The findings indicated that the pooled NTM prevalence rate was 0.158 (95% CI: 0.067-0.328), with a large I^2^ value (97.23%), reflecting a variable burden in terms of geography and other factors. A significant proportion of the studies were from countries such as China, Israel, Egypt and Taiwan. This may indicate that there are regional differences in the populations' environmental exposure to NTM and/or the diagnostic methods used [39].

The most common type of NTM species isolated from bronchiectasis patients was MAC, with a prevalence of 0.094 (95% CI: 0.045-0.185). In contrast, species such as *M. kansasii* presented a much lower frequency (0.008, 95% CI: 0.005-0.012), indicating variable pathogenic potential among various NTM species. The different ecological niches that each NTM species occupy and the different ways that these species interact with host factors to increase susceptibility to infection can be used to explain this variation. Higher exposure levels may affect the prevalence of these infections in bronchiectasis patients because NTM can survive in both soil and water [40,41].

Our comprehensive meta-analysis revealed an overall NTM and NTM-PD incidence of 15.8% (95% CI: 6.7%, 32.8%). The analysis demonstrated considerable heterogeneity, as reflected by elevated Q values and I^2^ indices, suggesting that the results exhibited substantial variation across different studies, although the prevalence estimates were similar. This finding is consistent with results from other studies [2,5,42]. For example, Zhou et al., in a systematic review and meta-analysis, reported a global prevalence of NTM in adults with non-CF bronchiectasis of approximately 10%, revealing substantial geographical differences largely affected by differences in local healthcare systems and environmental conditions [5]. In a related study, Chu et al. reported a 9.3% prevalence of NTM in patients with bronchiectasis, confirming high rates and highlighting the influence of methodological and geographic factors on prevalence figures [2]. Moreover, they found that the pooled prevalence was 7.7%, indicating difficulty reaching consensus due to significant regional heterogeneity [42].

Furthermore, Zhu et al. found that the pooled prevalence of NTM in patients with bronchiectasis was 7.7%. This finding highlights the difficulty in reaching a consensus on prevalence rates due to significant regional heterogeneity [42].

Our research indicated that the MAC was the most commonly isolated species in bronchiectasis patients. This finding is consistent with previous studies that recognized MAC as the main species involved in NTM infections within similar patient populations. In particular, Tan et al. reported significant proportions of both MAC and *M. abscessus* in their investigations performed in southern China [43]. In addition, Zhou et al. supported this finding by identifying MAC as the dominant subspecies in their studied population [5].

Máiz et al. reported differences in NTM frequency between different cohorts, which may be due to factors such as geographic diversity or methodological differences [44]. Additionally, a study by Schildknecht et al. concentrated on the elderly population with bronchiectasis, demonstrating that older age may increase susceptibility to NTM, a notion that is consistent with our analysis, which included participants from a range of age groups [45].

The current study had a number of shortcomings. There is a greater chance of publication bias because the meta-analysis was restricted to published papers. The substantial heterogeneity between the research may have been influenced by the populations' diversity. Patients' symptoms and problems were not sufficiently explained.

## 5 Conclusion

In conclusion, the findings of this systematic review highlight the increasing importance of NTM, especially the MAC, as significant pathogens in bronchiectasis. Due to the complexity of the disease and the multitude of factors that contribute to its progression, standardized diagnostic guidelines and increased clinical awareness are imperative. Future studies should examine the epidemiological characteristics of NTM infections in patients with bronchiectasis, as well as their impact on lung health, quality of life, and treatment outcomes across different populations. This review's robustness is due to the thorough search and data collection.

## Funding declaration

No funding

## CRediT authorship contribution statement

**Mosayeb Rostamian:** Conceptualization, Methodology, Software. **Shirin Bavari:** Data curation, Writing – original draft. **Nabi Jomehzadeh:** Investigation, Visualization. **Maryam Haddadzadeh Shoushtari:** Conceptualization, Investigation. **Ebrahim Kharazinejad:** Data curation, Writing – original draft. **Amir Zahedi:** Software, Validation. **Arezo Barani:** Data curation, Methodology. **Milad Abdi:** Data curation, Investigation. **Sara Kooti:** Supervision, Writing – review & editing.

## Declaration of competing interest

The authors declare that they have no known competing financial interests or personal relationships that could have appeared to influence the work reported in this paper.
