# Supplementary material for: Prevalence of nontuberculous mycobacteria in bronchiectasis: A systematic review and meta-analysis
Source: New Microbes New Infect. 2026 Jun 6;72:101781. doi: 10.1016/j.nmni.2026.101781 (PMC13262173; doi:10.1016/j.nmni.2026.101781)
Supplement: Multimedia component 1 [file mmc1.docx]

**Supplementary Table S1**. Detailed search strategy for each database

| **Database** | **Search date** | **Search strategy** |
| --- | --- | --- |
| PubMed | 2024   November  21 | (((atypical Mycobacteria[Title/Abstract]) OR (nontuberculous Mycobacteria[Title/Abstract])) OR (NTM[Title/Abstract])) AND (bronchiectasis[Title/Abstract]) |
| Scopus | 2024   November  21 | (((“atypical Mycobacteria”[Title/Abstract/keywords]) OR (“nontuberculous Mycobacteria”[Title/Abstract/ keywords])) OR (NTM[Title/Abstract/ keywords])) AND (bronchiectasis[Title/Abstract/ keywords]) |
| Web of Science | 2024   November  21 | (((“atypical Mycobacteria”[Title]) OR (“nontuberculous Mycobacteria”[Title])) OR (NTM[Title])) AND (bronchiectasis[Title]) |
